# Assessment of the emotional responses produced by exposure to real food, virtual food and photographs of food in patients affected by eating disorders

**DOI:** 10.1186/1744-859X-9-30

**Published:** 2010-07-05

**Authors:** Alessandra Gorini, Eric Griez, Anna Petrova, Giuseppe Riva

**Affiliations:** 1 Applied Technology for Neuro-Psychology Laboratory, Istituto Auxologico Italiano IRCSS, Milan, Italy; 2 Research Institute Brain and Behaviour, Maastricht University and Academic Anxiety Center, Maastricht, The Netherlands; 3 Faculty of Psychology, Moscow State University, Moscow, Russia; 4 Faculty of Psychology, Università Cattolica del Sacro Cuore, Milan, Italy

## Abstract

**Background:**

Many researchers and clinicians have proposed using virtual reality (VR) in adjunct to *in vivo *exposure therapy to provide an innovative form of exposure to patients suffering from different psychological disorders. The rationale behind the 'virtual approach' is that real and virtual exposures elicit a comparable emotional reaction in subjects, even if, to date, there are no experimental data that directly compare these two conditions. To test whether virtual stimuli are as effective as real stimuli, and more effective than photographs in the anxiety induction process, we tested the emotional reactions to real food (RF), virtual reality (VR) food and photographs (PH) of food in two samples of patients affected, respectively, by anorexia (AN) and bulimia nervosa (BN) compared to a group of healthy subjects. The two main hypotheses were the following: (a) the virtual exposure elicits emotional responses comparable to those produced by the real exposure; (b) the sense of presence induced by the VR immersion makes the virtual experience more ecological, and consequently more effective than static pictures in producing emotional responses in humans.

**Methods:**

In total, 10 AN, 10 BN and 10 healthy control subjects (CTR) were randomly exposed to three experimental conditions: RF, PH, and VR while their psychological (Stait Anxiety Inventory (STAI-S) and visual analogue scale for anxiety (VAS-A)) and physiological (heart rate, respiration rate, and skin conductance) responses were recorded.

**Results:**

RF and VR induced a comparable emotional reaction in patients higher than the one elicited by the PH condition. We also found a significant effect in the subjects' degree of presence experienced in the VR condition about their level of perceived anxiety (STAI-S and VAS-A): the higher the sense of presence, the stronger the level of anxiety.

**Conclusions:**

Even though preliminary, the present data show that VR is more effective than PH in eliciting emotional responses similar to those expected in real life situations. More generally, the present study suggests the potential of VR in a variety of experimental, training and clinical contexts, being its range of possibilities extremely wide and customizable. In particular, in a psychological perspective based on a cognitive behavioral approach, the use of VR enables the provision of specific contexts to help patients to cope with their diseases thanks to an easily controlled stimulation.

## Background

In the last few years there have been many attempts to treat mental disorders using virtual reality (VR), an innovative technique that allows patients to virtually experience critical situations (for example, exposure to a phobic stimulus) in a very safe environment while under the direct supervision of their therapists (for recent reviews see [1-3]). Following a cognitive behavioral-based approach, therapists can take advantage of interactivity and flexibility offered by virtual environments to measure and monitor a wide variety of patients' responses in real time, overcoming the limitations usually encountered during the *in vivo *exposure. Differently from what happens in real life settings, virtual environments can be tailored to the patients' needs and/or to therapeutic scopes in order to create specific and highly controlled exposure settings. Moreover, compared to the most used therapeutic approaches, such as guided imagination or exposure to photographic materials, VR allows subjects to interact and manipulate 3 D environments, mimicking interaction with objects in the real world. This experience increases the ecological validity of the simulated environment and enhances the 'sense of presence', defined as 'the user's sense of "being there" in the virtual environment' [4], or 'a perceptual illusion of non-mediation' [5]. In other words, the sense of presence is what happens when users 'forget' that their perceptions are mediated by technologies, feeling part of the virtual world 'as it was real' [6]. Through the increasing of the sense of presence, patients experience vivid real-life recreations that offer them contextual cues and facilitate generalization [7-9].

Today there is a growing recognition that VR may play an important role in clinical psychology, being a valid alternative to real-life exposure. However, the 'virtual approach' can be accepted only if real and virtual exposures elicit a comparable emotional response in subjects [10]. In order to verify whether virtual stimuli are as effective as real ones, and more powerful than static photographs, we assessed the emotional responses to real food (RF), virtual reality (VR) foods and photographs of food (PH) in two samples of patients affected, respectively, by anorexia (AN) and bulimia nervosa (BN), and in a sample of healthy controls (CTR). The reason why we chose food exposure is that, in addition to other situations of equal or more importance, it is one of the most typical conditions that provokes an emotional response in patients affected by eating disorders (ED) [11-14].

Various studies have used virtual stimuli instead of real ones to assess and treat eating behaviors in ED patients [15,16], but the first systematic attempts to evaluate the usefulness of virtual environments in provoking emotional reactions in such patients were carried out by Ferrer-Garcia *et al. *and Gutierrez-Maldonado *et al. *[17,18]. They created six virtual environments representing situations that are emotionally significant to subjects with eating disorders, and measured the level of state anxiety and depression in participants after exposure to each of them concluding that, upon simulation of real-life stressful situations, these environments are effective in producing significant emotional reactions in their users. Using a similar approach, but comparing the virtual stimuli directly with the real ones, and with their correspondent pictures, we wanted to test the psychological and physiological reactions to food in a sample of ED patients (half anorexic and half bulimic) and healthy controls. The two main hypotheses of the study were the following: (1) that the virtual exposure elicits emotional responses comparable to those produced by the real exposure, and (2) the sense of presence induced by the VR immersion makes the virtual experience more ecological, and consequently more effective than static pictures in producing emotional responses in humans.

## Methods

### Subjects

The experimental sample included 20 female patients affected by eating disorders (10 AN and 10 BN) and a control group of 10 healthy females (CTR) matched for age with the experimental groups. The mean body mass index (BMI) was 17.05 ± 1.09 in the AN group, 24.40 ± 4.05 in the BN group, and 21.82 ± 2.50 in the CTR group (see Table 1 for details). Patients were randomly recruited from the outpatient units of two public Italian hospitals in Milan, Italy, while CTR subjects were recruited through local advertisements among college students, administrative and workers' staff of the hospitals. Exclusion criteria for the AN and BN groups were the presence of lifetime psychiatric diseases other than eating disorders, major medical diseases, neurological syndromes, and brain injury or trauma. Consensus diagnoses, according to the *Diagnostic and Statistical Manual of Mental Disorders, fourth edition *(DSM-IV) criteria, were obtained by two clinicians who independently assessed all patients using a clinical interview and the Mini International Neuropsychiatric Interview Plus (MINI) [19], a diagnostic instrument designed to meet the need for a short but accurate structured psychiatric interview for DSM-IV and ICD-10 disorders. The severity of eating symptoms was then assessed with the Eating Disorders Inventory 2 (EDI-2) [20] (see Table 2 and the section on Psychological assessment for details). The MINI was also administered to the CTR group in order to exclude the presence of any psychiatric diseases, including actual or past eating disorders. Control subjects who were following a diet at the moment of the experiment were also excluded from the study.

**Table 1:** Age and body mass index (BMI) averages of control (CTR) and eating disorder (ED) groups

Group	Minimum	Maximum	Mean	SD
Control (N = 10)				

Age	19	34	26.20	5.14

BMI	18.01	25.80	21.82	2.50

ED (AN) (N = 10)				

Age	16	31	22.30	5.62

BMI	15	18.1	17.05	1.08

ED (BN) (N = 10)				

Age	17	32	23.90	5.26

BMI	18.45	30.60	24.40	4.05

AN = anorexia; BN = bulimia nervosa.

**Table 2:** Eating Disorders Inventory 2 (EDI-2) averages of anorexia (AN) and bulimia nervosa (BN) groups

	AN, mean (SD)	BN, mean (SD)
EDI-2		

Drive for thinness	9.13 (4.11)	12.15 (6.03)

Bulimia	3.01 (3.49)	9.13 (7.01)

Body dissatisfaction	13.05 (7.14)	18.41 (6.22)

Ineffectiveness	6.57 (5.09)	10.34 (5.67)

Perfection	5.66 (2.34)	3.1 (3.45)

Interpersonal distrust	6.70 (4.56)	5.50 (3.9)

Interceptive awareness	8.03 (5.67)	11.34 (8.43)

Maturity fears	4.23 (3.98)	6.23 (4.53)

Asceticism	3.56 (3.45)	5.89 (3.89)

Impulse regulation	4.34 (4.49)	7.03 (5.79)

Social insecurity	8.09 (5.89)	7.98 (6.35)

Subjects who gave their written informed consent to participate were included in the study. When participants were under 18, informed consent was obtained from their parents.

### Assessment

#### Psychological assessment

The following questionnaires were administered to the participants before the experiment.

##### EDI-2

EDI-2 [20], a self-report questionnaire that provides clinical information regarding the psychological and behavioral dimensions usually associated with anorexia and bulimia nervosa.

##### Stait Anxiety Inventory (STAI-S)

The STAI-S was initially conceived as a research instrument for the study of anxiety in adults. According to the author, state anxiety reflects a 'transitory emotional state or condition of the human organism that is characterized by subjective, consciously perceived feelings of tension and apprehension, and heightened autonomic nervous system activity'. State anxiety may fluctuate over time and can vary in intensity, in contrast with the trait anxiety that denotes 'relatively stable individual differences in anxiety proneness...' and refers to a general tendency to respond with anxiety to perceived threats in the environment [21]. Scores on the STAI-S have a direct interpretation: high scores mean more state anxiety and low scores mean less.

##### Visual analogue scale for anxiety (VAS-A)

The VAS-A [22] is a 100 mm vertical line with end points anchored as no anxiety at the bottom of the scale and anxiety as bad as it could possibly be at the top; scores range from 0 to 10. Among the numerous tools available for assessing anxiety, direct scaling procedures, such as the VAS, are popular because of their simplicity, versatility, relative insensitivity to bias effects, and the assumption that the procedures yield numerical values that are valid, reliable, and on a ratio scale [23-25].

##### ITC-Sense of Presence Inventory (ITC-SOPI)

The ITC-SOPI [26] is a validated questionnaire focusing on users' experiences of virtual reality (and media, in general) that evaluates the degree to which the subject experienced the 'sense of being in the virtual environment', how far the virtual environment was the dominant reality, and how far it is recalled as a 'place'.

#### Psychophysiological assessment

The Biograph Infiniti (Thought Technology Ltd, New York, USA) biofeedback equipment was used to measure the heart rate (HR) and respiration rate (RESP), and the skin conductance (SCR) of subjects before (baseline) and during exposure to food.

#### Experimental procedures

All subjects were presented to the following three conditions, outlined below.

##### Condition 1: real food view (RF)

**Figure 1:**
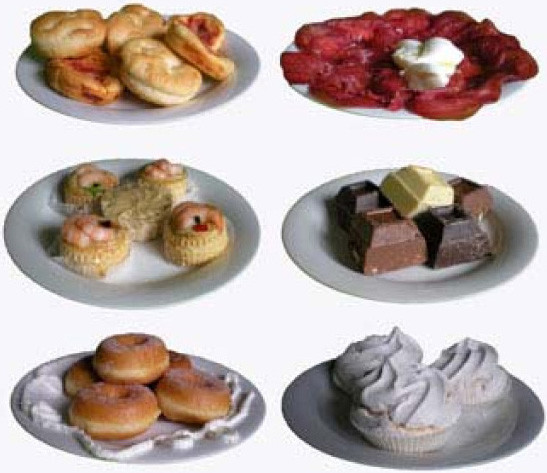
**The six high-calorie foods (three savory and three sweet foods) presented to the subjects in the three experimental conditions**.

Six real high-calorie foods (three savory and three sweet) (Figure 1) were presented for 30 s each with a pause of other 30 s between each other on a table in front of the subject. During the pause, all foods were covered with six red plastic lids so that subjects could not see them.

##### Condition 2: photograph slide show (PH)

A slideshow presentation including the photographs of the same six foods presented in the RF condition was presented on a computer screen. The presentation time and the interval between the different pictures were the same used in the RF condition. During the 30 s pauses a picture of the red lid covering a hidden food appeared on the screen.

##### Condition 3: virtual reality (VR) immersive condition

**Figure 2:**
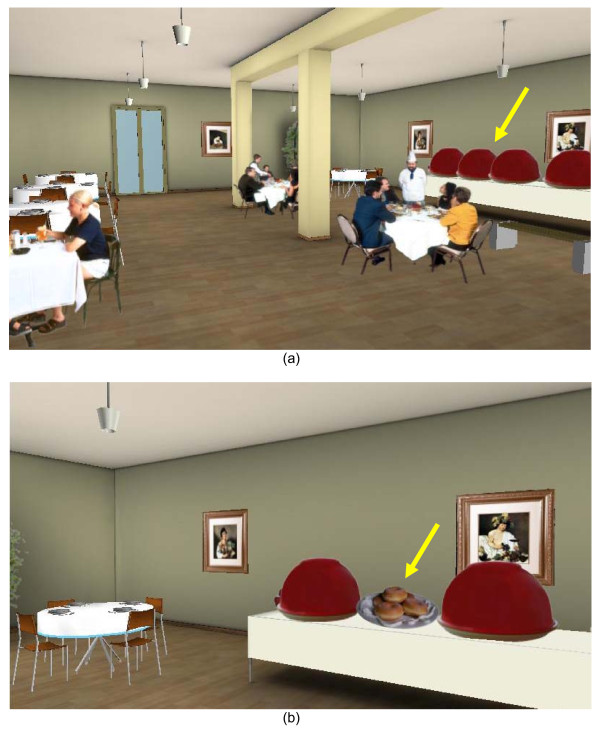
**The virtual reality (VR) restaurant**. **(a) **In the VR condition, subjects were asked to move around the room and to stand in front of the six plates covered by the red lids (the same used in the real food (RF) and photograph (PH) conditions) indicated by the yellow arrow on the right side of the figure. **(b) **Standing in front of the plates, subjects were asked to select them one by one, to virtually remove the lid and to observe the food for 30 s. After this time, the lid was automatically put back on the plate and the subject could do the second selection.

In the VR condition subjects were asked to wear a head mounted display (HMD) in order to have a 3 D view of the virtual environment. The motion tracker included in the HMD and a joystick allowed them to explore the environment and to interact with the virtual food. The environment represented a small restaurant with a buffet table in it (the virtual restaurant is included in NeuroVR [27], free open source software available at http://www.neurovr.org). A virtual representation of the same six foods presented in the RF and PH conditions appeared on the restaurant table and subjects were asked to explore the environment and to virtually open the lids one by one observing the food for 30 s, as happened in the two other conditions (Figure 2).

The order of presentation of each experimental condition, as well as the order of appearance of each food within the different conditions, was counterbalanced for each participant following a previously established randomization schema obtained from http://www.randomizer.org/.

All subjects were tested at least 2 h after a meal in order to avoid effects related to excessive hunger or overeating.

Before the RF and PH conditions there was a 3 min baseline during which subjects were asked to stay completely relaxed, while their physiological parameters were recorded. Because in the VR condition subjects used their right hand (all participants were right handed) to move inside the environment using a joystick, in order to control the hand movement, the baseline for the VR condition was recorded during a virtual navigation through an empty neutral space.

**Figure 3:**
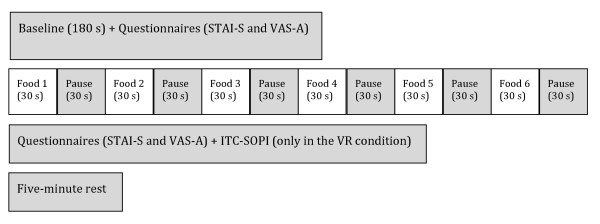
**Time schedule of the experiment (repeated for all the three conditions)**.

Once the physiological baselines were recorded, subjects were also asked to complete the STAI-S and the VAS-A. After that, the experimental session started, and heart rate, skin conductance and respiration rate were continuously recorded until the end of the task. Then, in order to measure the psychological variations occurred during the three different exposure conditions, subjects completed the STAI-S and the VAS-A again immediately after each session. The Presence Questionnaire was also administered at the end of the VR exposure. A pause of 5 min was planned between the sessions (Figure 3).

### Statistical analysis

Several within-subject repeated measure analysis of variance (ANOVA) tests were performed separately in each of the three groups of subjects to calculate the effects of exposure to the different kinds of food (real, photograph, and virtual) compared to the baseline. Then, the differences between each dependent variable measured after the exposure to food and the correspondent baseline were calculated. In the case of physiological measurements we calculated the differences between the mean values of HR, SCR and RESP recorded during the exposure and the mean values obtained from the correspondent 3 min baselines. These values were used to conduct several 3 × 3 repeated measure ANOVA tests in order to test whether participants' psychological and physiological responses changed depending on the kind of exposure (real food, pictures of food or virtual food), and the group (AN, BN or CTR). Finally, we calculated if symptoms severity, and the degree of presence experienced in the VR condition influenced the subjects' responses.

## Results

Within-subject repeated measure ANOVA tests showed that exposure to real food, photographs of food and virtual food caused a significant increase in the STAI-S questionnaire, VAS-A, HR and SCR in both AN and BN patients, but not in the respiration rate, compared to the baseline. However, no differences were found between the baseline and the three experimental conditions in the CTR group (Table 3).

**Table 3:** Within-subject repeated measure analysis of variance (ANOVA) tests comparing the effects of different types of food presentation (real food (RF), photographs (PH), virtual reality (VR)) on psychological and physiological responses of anorexia (AN), bulimia nervosa (BN) and control (CTR) subjects compared to the baseline (only significant values are reported)

	RF		PH		VR	
	
	F	*P *value	F	*P *value	F	*P *value
Psychological						

STAI-S						

AN	5.82	0.012	4.01	0.048	5.78	0.02

BN	5.12	0.025	3.52	0.04	5.01	0.029

VAS-A						

AN	5.01	0.018	4.1	0.045	4.98	0.03

BN	5.09	0.026	3.7	0.037	5.01	0.029

Physiological						

HR						

AN	4.49	0.031	4.2	0.043	5.01	0.029

BN	5	0.027	2.99	0.045	4.99	0.03

SCR						

AN	5.98	0.09	4.05	0.045	4.9	0.03

BN	3.2	0.038	2.28	0.048	4.8	0.033

HR = heart rate; SCR = skin conductance; STAI-S = Stait Anxiety Inventory; VAS-A = Visual Analogue Scale for anxiety.

### Variations in psychological responses depending on the kind of exposure in patients and controls

Repeated measures ANOVA tests were conducted in order to test whether the responses to the STAI-S and the VAS-A changed depending on the presentation condition (RF, PH, VR), and the group (AN, BN or CTR).

**Figure 4:**
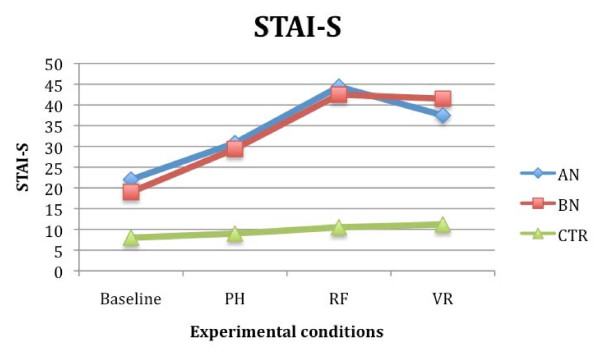
**Stait Anxiety Inventory (STAI-S) mean scores in anorexia (AN), bulimia nervosa (BN) and control (CTR) groups after the three different food exposures**.

**Figure 5:**
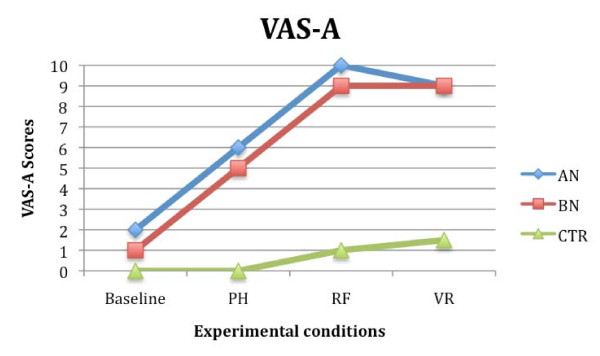
**Visual Analogue Scale for anxiety (VAS-A) mean scores in anorexia (AN), bulimia nervosa (BN) and control (CTR) groups after the three different food exposures**.

Results regarding the STAI-S showed a significant effect of the variables 'condition' (F (2,54) = 2.592; *P *< 0.05; partial eta^2 ^= 0.102) and 'group' (F (2, 27) = 1.89; *P *< 0.05; partial eta^2 ^= 0.099), and a significant interaction between them (F (4, 54) = 2.986; *P *< 0.05; partial eta^2 ^= 0.087). Similar results were obtained analyzing the VAS-A scores: the effect of the variables condition (F (2, 54) = 3.097; *P *< 0.05; partial eta^2 ^= 0.089) and group (F (2, 27) = 1.98; *P *< 0.05; partial eta^2 ^= 0.107), and the interaction between the variables condition and group were significant (F (4, 54) = 1.85; *P *< 0.05; partial eta^2 ^= 0.076). Post hoc analysis and contrasts showed that both AN and BN groups experienced higher level of subjective anxiety compared to the CTR subjects (*P *< 0.001), and that they felt significantly more anxious when exposed to real and virtual food than when they were exposed to the pictures of food (*P *< 0.05). No significant differences were found between the STAI-S and the VAS-A values recorded during real and virtual exposure in the two groups of eating disorder patients. CTR subjects showed similar STAI-S and VAS-A scores in all conditions (Figures 4 and 5).

### Variations in physiological responses depending on the kind of exposure in patients and controls

Repeated measures ANOVA tests (3 (conditions) × 3 (groups)) were also conducted in order to test whether HR, SCR, and RESP changed depending on the presentation condition (RF, PH, VR), and the group (AN, BN or CTR without ED).

**Figure 6:**
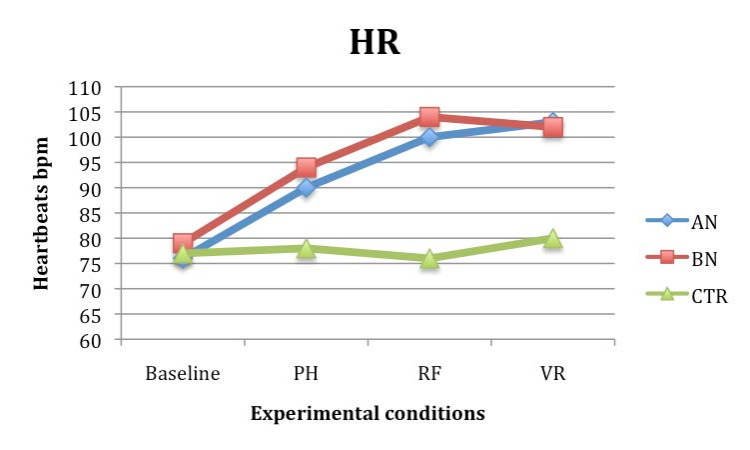
**Heart rate (HR) mean scores in anorexia (AN), bulimia nervosa (BN) and control (CTR) groups recorded during the three different food exposures**.

**Figure 7:**
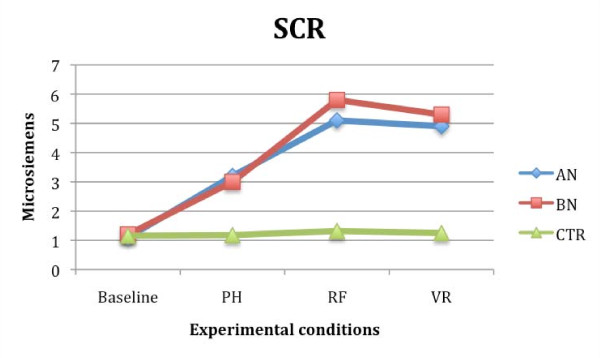
**Skin conductance (SCR) mean scores in anorexia (AN), bulimia nervosa (BN) and control (CTR) groups recorded during the three different food exposures**.

Results regarding the HR showed a significant effect of the variables condition (F (2,54) = 1.245; *P *< 0.05; partial eta^2 ^= 0.108) and group (F (2, 27) = 1.042; *P *< 0.05; partial eta^2 ^= 0.112), and a significant interaction between them (F (4, 54) = 2.002; *P *< 0.05; partial eta^2 ^= 0.083). Similar results were also obtained analyzing the SCR values. Once again, the effect of the variables condition (F (2, 54) = 2.438; *P *< 0.05; partial eta^2 ^= 0.065) and group (F (2, 27) = 1.98; *P *< 0.05; partial eta^2 ^= 0.086), and the interaction between the variables condition and group were significant (F (4, 54) = 1.322; *P *< 0.05; partial eta^2 ^= 0.075). Post hoc analysis and contrasts showed higher HR (*P *< 0.05) and SCR (*P *< 0.05) in AN and BN groups compared to CTR subjects. In both groups of patients, the level of physiological anxiety was higher in the RF and VR condition, than in the PH condition (*P *< 0.05). No significant differences were found between HR and SCR values recorded during real and virtual exposure in the two groups of eating disorder patients. CTR subjects showed similar scores in all conditions (Figures 6 and 7).

No significant effects were found analyzing the RESP responses in any of the experimental group.

Finally, we investigated if the degree of presence experienced in the VR condition and measured with the ITC-SOPI questionnaire, and symptoms severity, assessed with the EDI-2 influenced the patients' emotional responses. As suggested by Gutierrez-Maldonado *et al. *[17], we divided the ED samples (AN and BN) into quartiles and selected the first (25% with the lowest scores on the ITC-SOPI) and the fourth (25% with the highest scores). A simple effect of the degree of presence on the STAI-S (F = 2.80, *P *< 0.05) and the VAS-A (F = 2.51, *P *< 0.05) was found. However, we did not find any significant effect of the EDI-2 score on patient emotional reactivity.

## Discussion

This preliminary study was aimed at testing the theoretical assumption that a virtual experience elicits emotional responses comparable to those produced by real exposure. In addition, we also assumed that the sense of presence induced by the VR immersion makes the virtual experience more realistic, and consequently more effective than static pictures, in producing emotional responses in humans. In accordance with the first hypothesis, our data show that virtual food is as effective as real food, and more effective than photographs of food, in producing psychological and physiological responses in patients with ED, suggesting a possible advantage of using virtual stimuli instead of static pictures as an alternative to real stimuli to induce emotional reactions in subjects. This finding appears to be not specifically related to the diagnosis (AN or BN), as suggested by the fact that there were no significant differences in the emotional response recorded between the two groups of patients. Not even the severity of illness seems to influence the patients' reactions, as subjects with a mild, moderate or severe eating disorder did not significantly differ in their emotional responses to real or virtual food. However, we did not find any significant variation in the controls' emotional reactions in any of the experimental conditions. This is not surprising because, as happens in real life, food does not represent a stressful stimulus for healthy people.

Regarding our second hypothesis, we found an effect of subjects' degree of presence experienced in the VR condition on their level of perceived anxiety (STAI-S and VAS-A): the higher the sense of presence, the higher the level of anxiety. The sense of presence in virtual reality is defined as 'the participant's sense of "being there" in the virtual environment' [4] and it is obtained through two factors: immersion and interaction. Immersion is provided by the use of technological devices such as HMDs that permit a 3 D experience, while interaction is the possibility given to the users to interact in real time with the virtual environment. The higher the sense of presence, the more realistic the virtual experience, and more intense the emotional involvement. Immersion and interaction are the key distinctive factors that make the difference between the VR and the PH conditions. In the latter, subjects can only passively observe static pictures, while in the VR condition they can actively explore the environment, approach the food and virtually touch it, as they would do in real-life situations. We argue that the effectiveness of virtual and real stimulations is the reason why both psychological (STAI-S, and VAS-A) and physiological (HR and SCR) responses appear to be consistently higher in the RF and VR than in the PH condition. Thus, this result showing a similar pattern of psychological and physiological responses is rather new considering that, to date, there have been many studies that separately investigated psychological or physiological responses during VR exposure, but only few assessing the effects of stressors presented in a virtual environment on the subjective and objective response of anxiety [28,29]. Regarding the general lack of significant variations on respiration, we hypothesize that it may be due to the fact that only respiration rate was assessed and not tidal volume, and anxiety mainly affects tidal volume rather than rate [30].

To date, despite the large amount of data demonstrating the efficacy of VR-based approaches for the treatment of different psychological disorders [2], none of the previous work had directly investigated if the exposure to virtual stimuli is able to elicit emotional reactions similar to those elicited by real-life exposure, which is the added value of using VR instead of simple static pictures. Even though it was accomplished on only two small samples of ED patients, these preliminary data encourage the use of VR in clinical (exposure therapy) and even non-clinical (task learning) settings in which a highly customizable and controllable stimulation is preferred to a real-life one. Additionally, our data emphasize the role of presence in the emotional processes, proving that, even if definitively more expensive, VR is preferable to static images for generating affective responses in humans. So, in accord with the previous studies [17,18], the present research adds some evidence that virtual stimuli can be used instead of the real ones to elicit patients' emotions.

Despite the clearness of the present findings, this study has some important limitations. First, the small number of subjects per group makes us cautious about the generalization of the results. A future randomized controlled study including a larger sample will address this issue. Second, in the VR condition subjects were exposed to virtual food in a virtual restaurant, while in the other two conditions they were exposed to food only. A restaurant is a broader stimulus than food because it elicits a complex context possibly inducing a greater level of anxiety than food alone, and also other fears, not strictly or necessarily related to food (for example, agoraphobia). In order to control this aspect in future studies, virtual food could be presented in neutral virtual environments not specifically related to eating contexts. Thus, even if considered a limitation in the present study, the possibility to measure subjects' reactions in a complex virtual environment is a great advantage offered by virtual reality, with poor feasibility for testing the subjects' responses in a real complex environment such as a restaurant.

## Conclusions

In conclusion, though preliminary, the present data show that virtual stimuli are as effective as real ones, and more effective than static pictures, in generating emotional responses in ED patients. Unlike exposure to photographs, *in vivo *exposure and guided imagination, VR offers a good ecological validity, and also a fair internal validity, while allowing strict control over the variables. More generally, the present results provide initial evidence of the potential of VR in a variety of experimental, training and clinical contexts, its range of possibilities being extremely wide and customizable. In particular, in a therapeutic perspective based on a cognitive behavioral approach, the use of VR instead of real stimuli facilitates the provision of very specific contexts to help patients to cope with their conditions through a very controlled stimulation. At the same time, the results of the present study indicate that even very low cost VR software like NeuroVR can be used to screen, evaluate, and eventually treat the emotional reactions provoked by specific stimuli in patients affected by psychological conditions.

## Competing interests

The authors declare that they have no competing interests.

## Authors' contributions

AG contributed to the conception and design of the study, was involved in drafting the manuscript, analyzing the data, and revising the text critically for intellectual content and was also involved in training the data collectors. EG participated in designing the study, and drafting and editing the manuscript. AP participated in acquisition, analysis and interpretation of data and was involved in drafting the manuscript. GR participated in drafting and editing the manuscript.
